# Optimization and modelling of magnesium oxide (MgO) photocatalytic degradation of binary dyes using response surface methodology

**DOI:** 10.1038/s41598-024-56797-6

**Published:** 2024-04-24

**Authors:** Hyeong Kwang Benno Park, Pushpendra Kumar, Imen Kebaili, Imed Boukhris, Yun Hwan Joo, Tae Hyun Sung, Anuruddh Kumar

**Affiliations:** 1 Department of Electrical Engineering, Hanyang University, Seoul, 04763 South Korea; 2 Indian Institute of Technology Mandi, Mandi, Himachal Pradesh 175005 India; 3 Department of Physics, Faculty of Science, King Khalid University, P.O. Box 9004, Abha, Saudi Arabia; 4 Center for Creative Convergence Education, Hanyang University, Seoul, 04763 South Korea

**Keywords:** Magnesium oxide (MgO), Photocatalysis, Binary dye, Parametric study, Parameter optimization, Central composite design (CCD), Response surface methodology (RSM), Environmental sciences, Materials science

## Abstract

Textile industry dye effluent contains a mixture of different kinds of dyes. Many times, photocatalysis is targeted as a solution for the treatment of dye effluent from the textile industry. Many researches have been published related to the photocatalysis of single textile dyes but in the real-world scenario, effluent is a mixture of dyes. Magnesium oxide (MgO) is used as a photocatalyst to treat a mixture (binary solution) of Methylene blue (MB) and Methylene violet (MV) along with individual MB and MV dyes in this article. MgO shows remarkable photocatalytic activity at about 93 and 88% for MB and MV dye in binary solution within 135 min. Furthermore, to study the influence of process parameters, experiments are designed with the help of the central composite design (CCD), and Response surface methodology (RSM) is used to study the interactions between parameters. For this study, five parameters are selected i.e., Photocatalyst dosage, initial concentration of both dyes, time of exposure to the light source, and pH of the binary solution. The photocatalytic process is also optimized and finally optimization of process parameters is validated with an experiment. The result of the validation experiment is very close to the predicted photocatalytic activity.

## Introduction

Textile industries are using dyes in their process production. These dyes are very toxic to the environment ecosystem^1,2^. Textile industries discharge wastewater effluent directly into the environment. These wastewater effluents contain complex structured compounds that are difficult to biodegrade. These complex structured organic substances are toxic which makes dyes a threat to the human body. Researchers have developed some methods to tackle this problem. Methods are adsorption^3^, coagulation^4,5^, flocculation^6^. Photocatalysis is also a method that uses a photocatalyst material that is activated in the sunlight. These materials are stable, abundant, safe, and highly preferred as they are sustainable, non-hazardous, and economical. Metal oxides have been used in various applications such as electronics, catalysts, and biomedicine^7,8^. Many metal oxides (eg., TiO_2_^8^, ZnO^9^, Fe_2_O_3_^10^, and WO_3_^11^) have properties such as nontoxicity, high activity, chemical stability, and low cost and have been used as a photocatalyst material for dye degradation. Catalytic mechanisms of these materials have also been documented^12^. Nowadays magnesium oxide (MgO) has caught attention in various applications such as catalytic^13^, electronics^14^, and biomedical^15^ because of its unique physicochemical properties, low toxicity, and inexpensive properties. The application of MgO is very widespread in various fields such as adsorption^11^, catalysis^16^, antibacterial material^17^, paint^18^, and superconductors^19^. Researchers have made some attempts^13,14,20–24^ to use MgO as a photocatalyst material for the degradation of various dyes in aqueous solution.

Organic complex structured compounds such as methylene violet (MV), methyl orange (MO), methylene blue (MB), and rhodamine B (RhB) are the most hazardous and important dyes effluent from the textile industry^25–28^. Most of the researchers use one of these dyes to evaluate the photocatalysis process. But more importantly, in the real-world scenario effluent from the textile industry contains a mixture of dyes so a mixture of dyes is more relevant to consider for photocatalysis. Some of the researchers have tried a mixture of dyes (binary dye) to evaluate the photocatalysis activity by using different combinations of dyes e.g. MO/RhB^29^, MO/MB^29,30^, and MB/RhB^31^. Furthermore, one thing is to be noted the textile industry effluent contains a mixture of dyes with different individual dye concentrations at variable pH. So, this concentration and pH of effluent are also important parameters to consider while designing any catalyst material. The amount of catalyst and time of catalysis are the other important parameters because they are directly associated with the effectivity of photocatalysts.

Conventionally, these process parameters are analyzed by holding a parameter constant while varying other parameters. The holding of a parameter at an unspecified constant level does not show the combined effect of all the parameters involved. This method is very time-consuming and requires a lot of experiments to optimize all parameters. By together optimizing all the influencing parameters using statistical experimental design, such as Response Surface Methodology (RSM)^7^, these limitations of the conventional technique can be avoided. Using statistical experimental design approaches in the development of photocatalytic processes can lead to several benefits, including improved photocatalytic activity, reduced process variability, closer confirmation of the output response to nominal and target requirements, and reduced development time and overall costs. Many researchers have used this Response Surface Methodology a statistical experimental design approach to optimize and understand the behavior of process parameters^32,33^. It is well well-established method and widely reported for photocatalysis experiments. However, it has not been explored for MgO as a photocatalyst for the degradation of binary dye.

This research article is related to the use of Magnesium Oxide (MgO) as a photocatalyst material for the degradation of a mixture of dyes (Binary dye). A mixture of Methylene Blue (MB) and Methyl Violet (MV) is used for this study because a mixture of dyes is more relevant in the case of textile effluent. Furthermore, the effect of process parameters is also analyzed and optimized with the help of Response Surface Methodology (RSM). Five parameters (Factors) such as pH of the solution, irradiation time, catalyst dosage, initial MB dye concentration, and initial MV dye concentration are selected for this study. To analyze these parameters, the experiments are designed with the help of Central Composite Design (CCD) by Design Expert (version 13) software. The quality of fitting of the model and the effect of individual and interaction of parameters are evaluated by analysis of variance (ANOVA). The parameters are optimized for the highest photoactivity and also validated by experiment.

## Material and methods

Magnesium oxide (MgO) powder was purchased from Adnano Technologies Pvt. Ltd., India. The absorption spectra of MB and MV dyes were collected using the Shimadzu UV-2600 UV–Vis spectrophotometer. MgO photocatalyst was drop cast in ethanol on a silicon wafer and Scanning electron microscope (SEM) images of MgO powder were recorded using FE-SEM Inspect™S50.

The photocatalysis experimental setup contains a visible light source, a reaction beaker, a magnetic stirrer, and a box. The reaction beaker was kept on a magnetic stirrer and a visible light source was placed above 15 cm from the upper surface of the dye solution. The visible light source was from Havells company and of 15 W power rating. The whole experimental setup was kept inside a box to avoid any type of unwanted radiation.

All the experiments were conducted in a 20 ml solution of dyes of known initial concentration, photocatalyst dosage, and pH. The pH of the dye solution was adjusted by 0.1 M HCl and 0.1 M NaCl solution. The dye aqueous solution was put in the dark for 5 h to establish the adsorption equilibrium of MB, and MV dyes on the photocatalyst surface area. After establishment, the dye solution was centrifuged at 3000*rpm* for 3 min to separate the photocatalyst and then analyzed by UV-visual spectrophotometer. Dye along with the photocatalyst used for analysis was again mixed with the dye solution. The dye solution was exposed to a light source. After the specified period dye solution was brought out again and was centrifuged at 3000*rpm* for 3 min. After this process, the mixture was again analyzed and subsequently, degradation efficiency was calculated via the following Eq. (1).

1 $${\text{P}}\, = \,\left( {{\text{Co}} - {\text{Ct}}} \right)/{\text{Co}}\, \times \,{1}00$$

    Where P is the degradation efficiency (%), Co is the initial concentration of the dye solution and Ct is the concentration of dye after a specific period.

The photocatalytic activity was evaluated for individual MB and individual MV dyes and a mixture of both dyes (binary dye). The initial concentration of single MB and MV dyes was 10 mg/L at natural pH and the amount of photocatalyst (MgO) used was 5 g/L. Photocatalytic activity was also evaluated for the binary mixture of MB and MV dyes. The volumetric ratio of MB and MV dyes was taken as 1:1 with 10 mg/L initial concentration of each dye. The amount of photocatalyst used was 5 g/L.

Interaction between given parameters (factors) was studied by RSM and experiments were designed by central composite design (CCD) with the help of Design Expert version 13 software. A CCD with five levels and five factors was selected. These factors were X1: Photocatalyst dosage (g/L), X2: time of exposure (minutes), X3: MB dye initial concentration (mg/L), X4: MV dye initial concentration (mg/L), and X5: pH of the dye solution. CCD can evaluate a quadratic model recognize the main effective factors interact them with a minimum number of required experiments and optimize multiple variables^34,35^. The ranges of factors along with the low, center, and high points can be seen in Table 1.

**Table 1:** Factors and their ranges for CCD matrix.

S.No	Factors	Unit	Low	Centre	High
1	X1: Photocatalyst dosage	g/L	3	5	7
2	X2: time of exposure	min	75	135	195
3	X3: MB dye initial concentration	mg/L	5	10	15
4	X4: MV dye initial concentration	mg/L	5	10	15
5	X5: pH		3	5	7

The CCD design suggested 27 individual experiments in two blocks and a small design. It is shown in Table 2. The polynomial equation [(Eq. 2)] was used for the mathematical relationship between independent parameters^36–38^. Analysis of variance (ANOVA) was used to evaluate the quality of the fitted model. The significance of the model was determined by p-value and f-value^39^.

2 $$Log_{10} (R) = \beta_{0} + \sum\nolimits_{i = 1}^{6} {\beta iXi} + \sum\nolimits_{i,j = 1}^{6} {\beta ijX1X2} + \sum\nolimits_{i = 1}^{6} {\beta iiXi2} + \varepsilon$$

**Table 2:** CCD matrix and responses.

Run	Block	Factors					Responses	
		X1: Photocatalyst dosage (g/L)	X2: Time (min)	X3: Initial MB concentration (mg/L)	X4: Initial MV concentration (mg/L)	X5: pH	R1: MB dye degradation efficiency (%)	R2: MV dye degradation efficiency (%)
1	1	5	135	10	10	6	77	82
2		7	75	5	15	8	48	52
3		3	195	5	15	8	77	92
4		3	195	15	5	8	57	67
5		5	135	10	10	6	78	82
6		7	195	15	5	4	70	73
7		5	135	10	10	6	79	83
8		7	195	5	15	4	77	56
9		7	75	15	5	8	21	26
10		7	195	5	5	8	67	62
11		5	135	10	10	6	70	77
12		3	195	15	15	4	58	75
13		3	75	5	5	4	53	56
14		3	75	15	15	8	22	45
15		7	75	15	15	4	28	42
16	2	5	135	10	10	6	67	73
17		5	135	10	19	6	70	85
18		5	135	10	10	9.6	71	78
19		1.3	135	10	10	6	6	24
20		5	135	19	10	6	40	53
21		8.6	135	10	10	6	71	75
22		5	135	10	10	2.3	66	77
23		5	135	10	10	6	56	68
24		5	135	0.9	10	6	98	83
25		5	26	10	10	6	6	8
26		5	244	10	10	6	88	89
27		5	135	10	0.9	6	75	77

Some experiments suggested by CCD (Table 2) showed combined degradation efficiency (MB and MV dyes) more than optimized values. While designing experiments by CCD, it is used to take some axial points beyond predefined ranges. Axial points are taken to observe results beyond predefined ranges and correlate them with experiments done in the range. Experiments run 24 and 26 are the axial points taken by CCD which are beyond predefined ranges. In an experiment run 24, a concentration of MB dye was taken for the axial point. As the concentration of MB dye was considerably less than the predefined range, it showed higher photodegradation efficiency than the predicted value. Experiment run 27 was also an axial point taken by CCD. CCD took time of reaction as an axial point. As the time of reaction was considerably higher than the predefined range, it showed higher photodegradation efficiency. Experiments 19 and 21 are also similar types of experiments.

## Results and discussion

**Figure 1:**
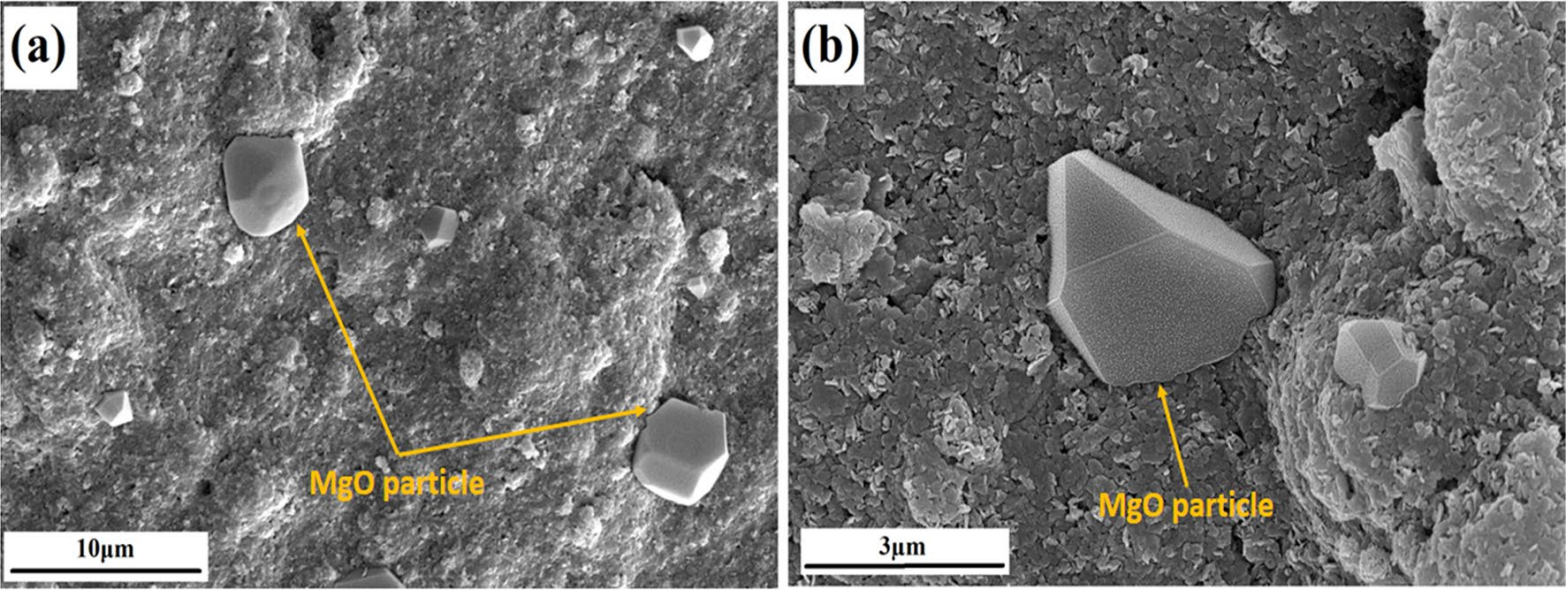
Scanning electron microscopy images of (**a**) a MgO particle and (**b**) a single particle of MgO.

Scanning electron microscope (SEM) images are shown in Fig. 1. The big particle shown in Fig. 1b is an MgO particle and the background is ethanol which was used for sample preparation (drop cast). It can be concluded from the images that MgO powder partials were in specific shapes and sizes. The average size of the powder particle was 2.5 μm. X-ray diffraction is not shown here as this material was purchased from the market and the same is directly used in the present experiments.

**Figure 2:**
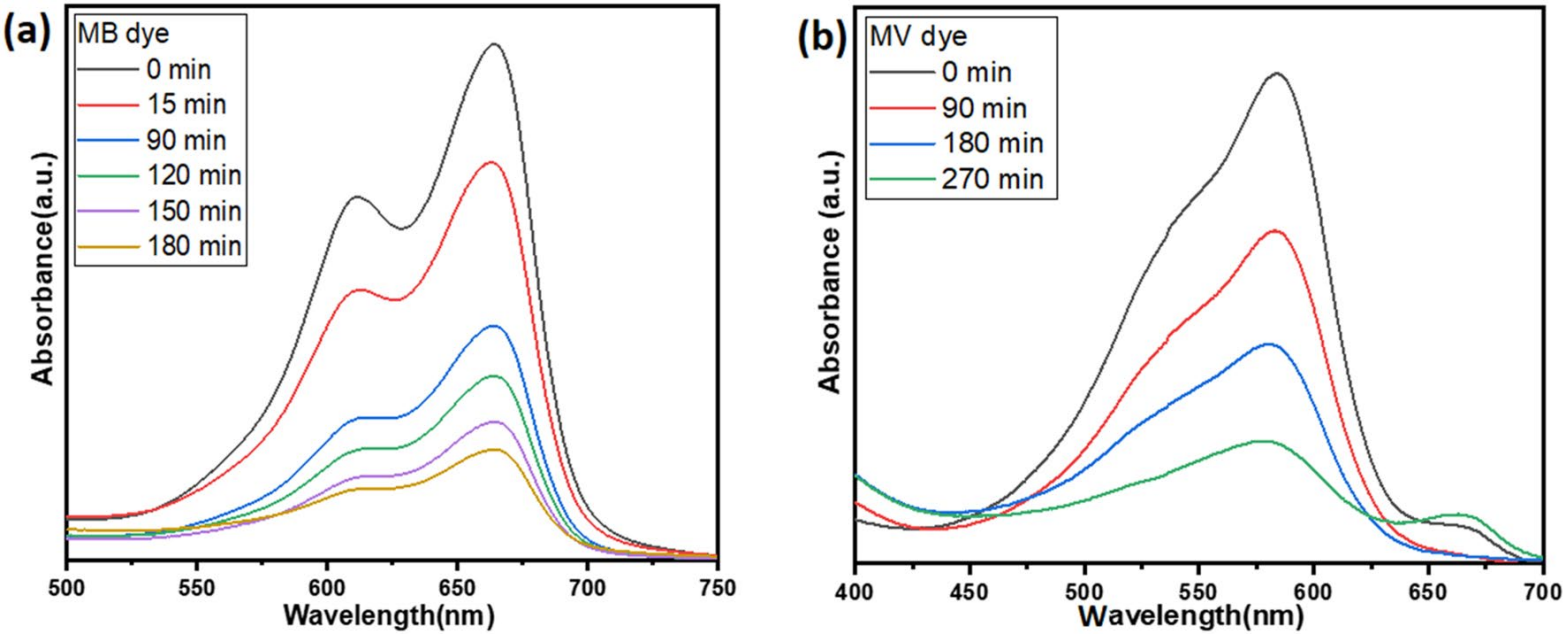
UV visible spectra of Photocatalysis of (**a**) MB dye and (**b**) MV dye at 5 g/L MgO powder dosage, 100 mg/L dye concentration, and natural pH.

The photocatalytic activity was evaluated for single MB and single MV dyes. For this purpose, the initial concentration of individual single MB dye and individual single MV dyes was 10 mg/L at natural pH and the amount of photocatalyst (MgO) was 5 g/L. MB and MV dye absorption characteristic peak was found at 663 and 587 nm respectively. The effect of photocatalysis was determined by monitoring characteristics peak. During the photocatalysis process, the intensity of the absorption peak was decreased which indicated a decrement in the concentration of dyes (Fig. 2). Figure 2a and Fig. 2b show the absorption Vs wavelength plot depicting this decrement for individual single MB and MV dyes respectively. MgO photocatalyst showed 78% degradation in 120 min when an individual single MB dye aqueous solution was used for photocatalysis (Fig. 2a). However, individual single MV dye showed an 87% degradation in 270 min (Fig. 2b). Many researches have been published to report the photocatalytic activity of MgO/modified MgO photocatalysts^40–45^.

**Figure 3:**
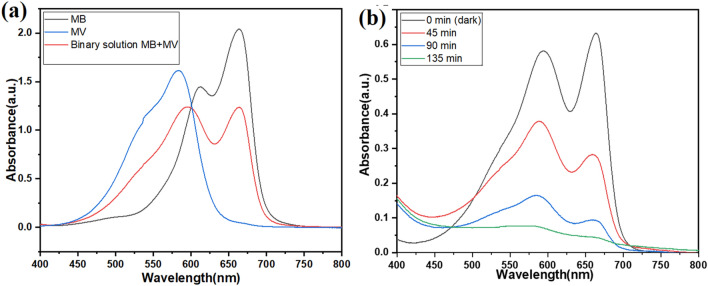
UV–visible spectra: (**a**) absorption spectra for a single and binary mixture of MB and MV dye (10 mg/L) and (**b**) absorption spectra for photocatalysis of 10 mg/L of each dye for 135 min, 5 g/L MgO nanoparticle dosage and natural pH.

The absorption Vs wavelength plot of individual single MB, single MV dye at a concentration of 10 *mg/L*, and binary solution of MB and MV dye with a volumetric ratio of 1:1 at a concentration of 10 mg/L are shown in Fig. 3a. It can be observed from the plot that after mixing both dyes in equal amounts (the volumetric ratio is 1:1), the intensity of the absorption peaks of binary dyes decreased. It indicated that the concentration of both dyes decreased. When two dyes were mixed, the amount of solvent increased because it came from both dyes. As the amount of solvent in the binary mixture of dyes increased and the amount of MB and MV dye was constant, the concentration of MB and MV dye in the binary mixture decreased.

The photocatalytic activity was evaluated for the binary mixture of MB and MV dyes. MB and MV dye absorption peaks were found at 663nm and 598 nm respectively. The effect of photocatalysis was determined by monitoring absorption characteristic peaks. During the photocatalysis process, the intensity of the absorption characteristics peak was decreased which indicated that the concentration of dyes was also decreasing (figure 3b). MB and MV dyes showed 93 and 88 % degradation efficiency in binary solution within 135 min respectively.

**Figure 4:**
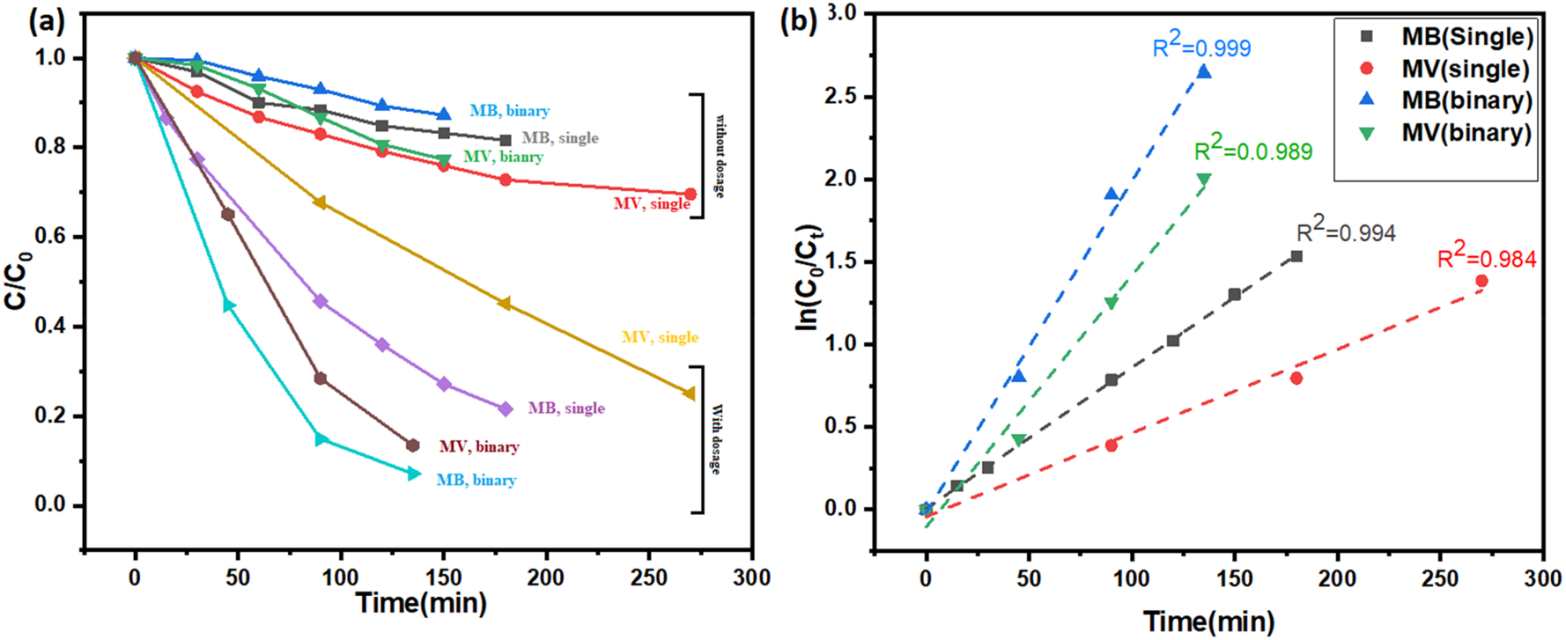
(**a**) C/Co versus time plots and (**b**) ln (Co/Ct) versus time plots for MB and MV single dyes and a mixture of both dyes.

Figure 4a shows the degradation efficiency of MgO photocatalyst (C/Co) versus time plots when an individual single MB and MV dyes and binary solutions of MB and MV dyes were used for photocatalysis. Figure 4a revealed that MB dye in a binary mixture of dyes is degrading fastest whereas individual single MV dye degrades slowest. Figure 4a also revealed that both dyes are degrading faster when they are in binary dye solution. It implies that when photocatalytic reactions of both dyes take place in the binary solution then both reactions help each other to increase the rate of reaction.

L–H equation has been often used for the modelling of photocatalytic degradation. It was expressed by a first-order kinetic model [Eq. (3)].

3 $${\text{ln }}\left( {{\text{C}}_{{\text{o}}} /{\text{C}}_{{\text{t}}} } \right)\, = \,{\text{kt}}$$

Here C_o_ is the initial concentration, C_t_ is the concentration of dye solution after the t time-period of light illumination for the photocatalytic experiment., and k (min^−1^) is the rate constant of the photocatalytic reaction. Empirical data was fitted for single MB and MV dye with and without photocatalyst along with MB and MV dyes in binary dye solution. Figure 4b shows (C_o_/C_t_) versus time graph with rate constant of reaction. The reaction rate constant for MB dye in the binary mixture of dyes is the highest whereas the same is the lowest in the case of single MV dye solution.

The electron (e^−^) and hole (h^+^) were generated by the ejection of electrons from the valance band to the conduction band when the MgO catalyst powder was illuminated. These electrons and holes migrate to the surface of the MgO catalyst powder particle and make an active site for advanced oxidation reactions. Free radicals are generated by the interaction of e^-^ and h^+^ with the H_2_O and O_2_ molecules at active sites. The HOO free radical was generated by the reduction reaction of superoxide anon (O_2_^−^). Now, the HOO free radical reacts with e^−^ and hole h^+^ and generates H_2_O and it consumes free energy and releases the hydroxyl radicals. Moreover, this h^+^ in the valance band reacts with H_2_O molecule and generates hydroxyl radicals^46^. These generated hydroxyl radicals attack on dye molecule break down the structure of the dye molecule and produce H_2_O and other byproducts^47^.

CCD was used to design experiments to study the influences of selected parameters (factors). The experiments along with their degradation efficiencies are shown in Table 2. The degradation activity and parameters fitted in Eq. (2) by software and analyzed by Analysis of variance (ANOVA) to evaluate the quality of the fitted equation. The significance of the fitting was determined by p-value and f-value. Table 3 illustrates the five‐factor CCD matrix and fitting results of the degradation of the binary dye solutions.

**Table 3:** ANOVA for a quadratic model with log_10_ transformation for photocatalysis of binary dye mixture.

Source	MB dye					MV dye				
	DF	SS	MS	F	P	DF	SS	MS	F	P
Block	1	0.0400	0.0400			1	0.0116	0.0116		
Model	16	2.52	0.1576	114.62	< 0.0001	17	1.37	0.0805	191.48	< 0.0001
X1	1	0.6369	0.6369	463.26	< 0.0001	1	0.1529	0.1529	363.53	< 0.0001
X2	1	0.9133	0.9133	664.26	< 0.0001	1	0.6461	0.6461	1535.87	< 0.0001
X3	1	0.1381	0.1381	100.44	< 0.0001	1	0.0230	0.0230	54.75	< 0.0001
X4	1	0.0038	0.0038	2.74	0.1323	1	0.0016	0.0016	3.82	0.0864
X5	1	0.0033	0.0033	2.44	0.1531	1	0.0000	0.0000	0.0665	0.8030
X1X2	1	0.1484	0.1484	107.91	< 0.0001	1	0.1048	0.1048	249.13	< 0.0001
X1X3	–	–	–	–	–	1	0.0024	0.0024	5.68	0.0444
X1X5	1	0.0331	0.0331	24.08	0.0008	1	0.0018	0.0018	4.21	0.0742
X2X3	1	0.0681	0.0681	49.50	< 0.0001	1	0.0078	0.0078	18.52	0.0026
X2X4	1	0.0168	0.0168	12.20	0.0068	1	0.0017	0.0017	3.98	0.0812
X3X4	1	0.2696	0.2696	196.11	< 0.0001	1	0.1517	0.1517	360.73	< 0.0001
X3X5	1	0.1330	0.1330	96.73	< 0.0001	1	0.0925	0.0925	219.82	< 0.0001
X4X5	1	0.2017	0.2017	146.74	< 0.0001	1	0.1399	0.1399	332.48	< 0.0001
X1^2^	1	0.3800	0.3800	276.40	< 0.0001	1	0.0858	0.0858	204.02	< 0.0001
X2^2^	1	0.3191	0.3191	232.10	< 0.0001	1	0.3324	0.3324	790.28	< 0.0001
X4^2^	1	0.0163	0.0163	11.83	0.0074	1	0.0094	0.0094	22.24	0.0015
X5^2^	1	0.0089	0.0089	6.51	0.0311	1	0.0052	0.0052	12.25	0.0081
Residual	9	0.0124	0.0014			8	0.0034	0.0004		
Lack of fit	5	0.0079	0.0016	1.41	0.3809	4	0.0023	0.0006	2.25	0.2259
Pure error	4	0.0045	0.0011			4	0.0010	0.0003		
Cor total	26	2.57				26	1.38			

To find the influencing factors and the interactions between factors, analysis of variance (ANOVA) was used^48–50^. In Table 3, p-values and f-values are indicated for individual coefficients in the equation. A p‐value less than 0.0001 shows the significant effect and statistical significance of results at a 95% confidence level. Whereas, the F-values for the model are 114.62 and 191.48 for MB and MV dye in binary solution respectively. The lack of fit (LOF) of the Fitted models for photodegradation of binary dye with MgO as photocatalyst is not significant which indicates the reliability of the fitted model and all values indicate that the model is perfectly fitted and can be used for further analysis.

**Figure 5:**
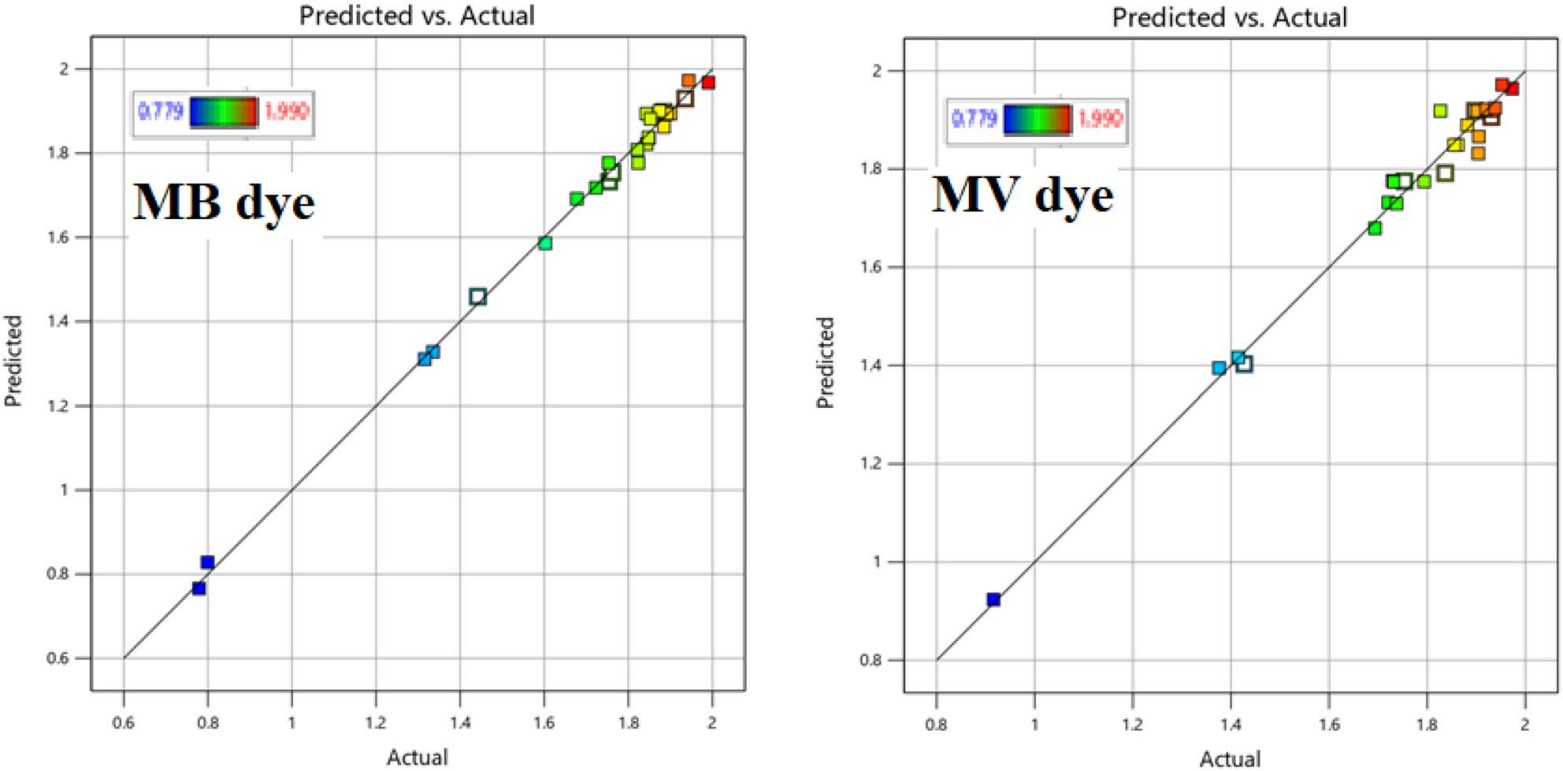
Predicted vs. actual photodegradation efficiency of (**a**) MB dye and (**b**) MV dye.

The values of adjusted and predicted R^2^ were evaluated (Table 4) and found around 0.98 and 0.83, and 0.99 and 0.90 for MB and MV dyes (respectively) in the binary solution. This is an indication of a good relation between the observed and predicted values. The signal‐to‐noise ratio (adequate precision) was 39.8 and 62.5 for MB and MV respectively. The final fitted equation for MB dye degradation is shown in Eq. (4) in terms of coded factors. Whereas, Eq. (5) shows the fitted equation for the degradation of MV in the binary solution in terms of coded factors.

4 $${\text{Log}}_{{{1}0}} \left( {{\text{R}}_{{1}} } \right)\, = \,{1}.{84}\, + \,0.{\text{2766X1}}\, + \,0.{\text{3312X2}}{-}0.{1}0{\text{48X3}}{-}0.0{\text{173X4}}\, + \,0.0{2}0{\text{1X5}}{-}0.{\text{1983X1X2}}{-}0.0{\text{937X1X5}}\, + \,0.{\text{1248X2X3}}\, + \,0.0{\text{619X2X4}}\, + \,0.{\text{2544X3X4}}\, + \,0.{\text{1744X3X5}}\, + \,0.{\text{2148X4X5}}{-}0.{\text{1341X1}}^{{2}} {-}0.{\text{1229X2}}^{{2}} \, + \,0.0{\text{277 X4}}^{{2}} \, + \,0.0{2}0{\text{6 X5}}^{{2}}$$

5 $${\text{Log}}_{{{10}}} \left( {{\text{R}}_{{2}} } \right)\,{ = }\,{1}{\text{.87}}\,{ + }\,{0}{\text{.1396X1}}\,{ + }\,{0}{\text{.2870X2{-}0}}{\text{.0435X3}}\,{ + }\,{0}{\text{.0143X4{-}0}}{\text{.0019X5{-}0}}{\text{.1759X1X2}}\,{ + }\,{0}{\text{.0241X1X3{-}0}}{\text{.0229X1X5}}\,{ + }\,{0}{\text{.0436X2X3{-}0}}{\text{.0222X2X4}}\,{ + }\,{0}{\text{.1925X3X4}}\,{ + }\,{0}{\text{.1502X3X5}}\,{ + }\,{0}{\text{.2032X4X5{-}0}}{\text{.0637 X1}}^{{2}} {\text{{-}0}}{\text{.1254 X2}}^{{2}} \,{ + }\,{0}{\text{.0210X4}}^{{2}} \,{ + }\,{0}{\text{.0156X5}}^{{2}}$$

where R_1_ and R_2_ are the degradation efficiency of MB and MV dyes in a binary mixture of dyes. Figure 5 illustrates the correlation between predicted and experimental MB and MV dye degradation efficiency. These plots show that all points are in a straight line and indicate a good correlation between predicted and actual values of degradation.

**Table 4:** Model fitting statistics.

Dye	Standard deviation	Mean	CV (%)	PRESS	R^2^	Adjusted R^2^	Predicted R^2^	Adequate precision
MB	0.0371	1.70	2.18	0.4125	0.9951	0.9864	0.8372	39.8467
MV	0.0205	1.78	1.15	0.1265	0.9975	0.9923	0.9079	62.5551

**Figure 6:**
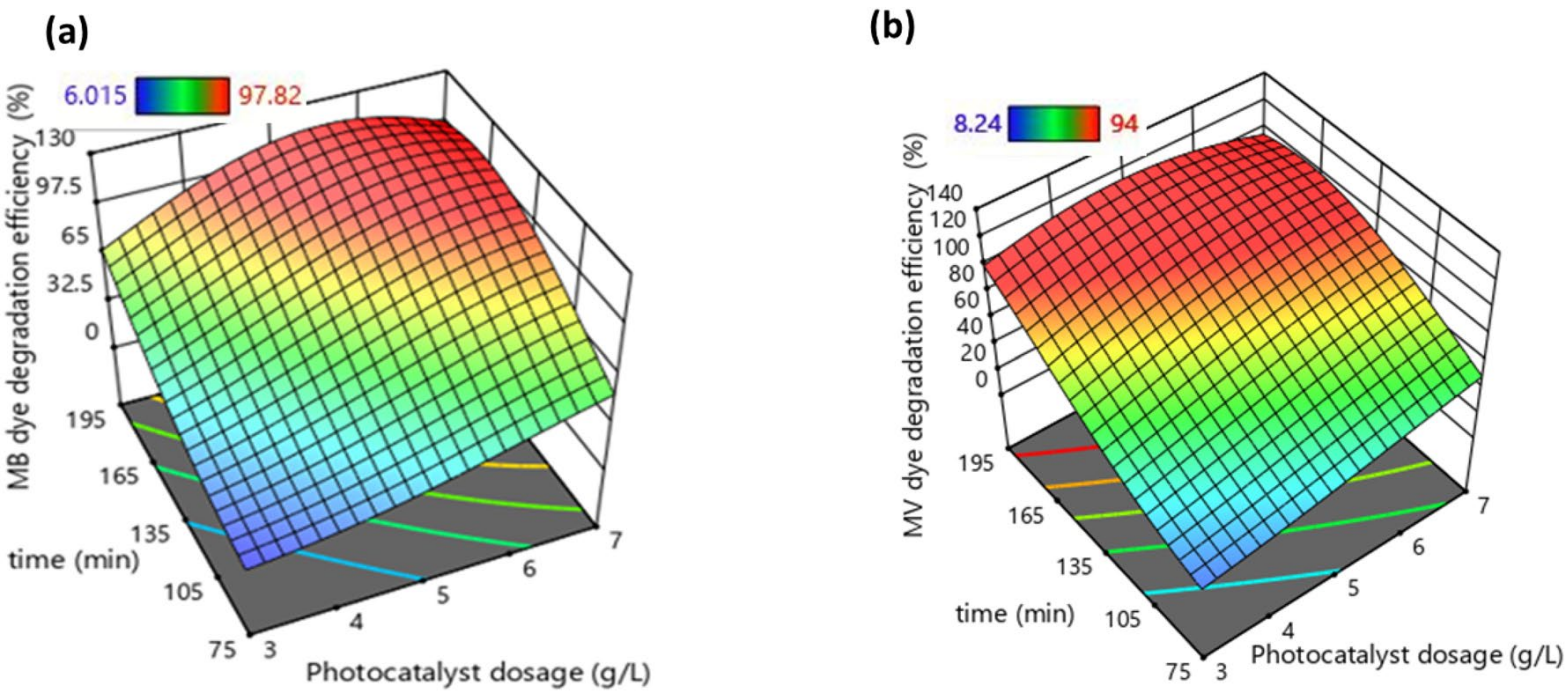
3D plots showing the influence of photocatalyst dosage and period of photocatalysis reaction on (**a**) MB dye and (**b**) MV dye degradation efficiencies.

**Figure 7:**
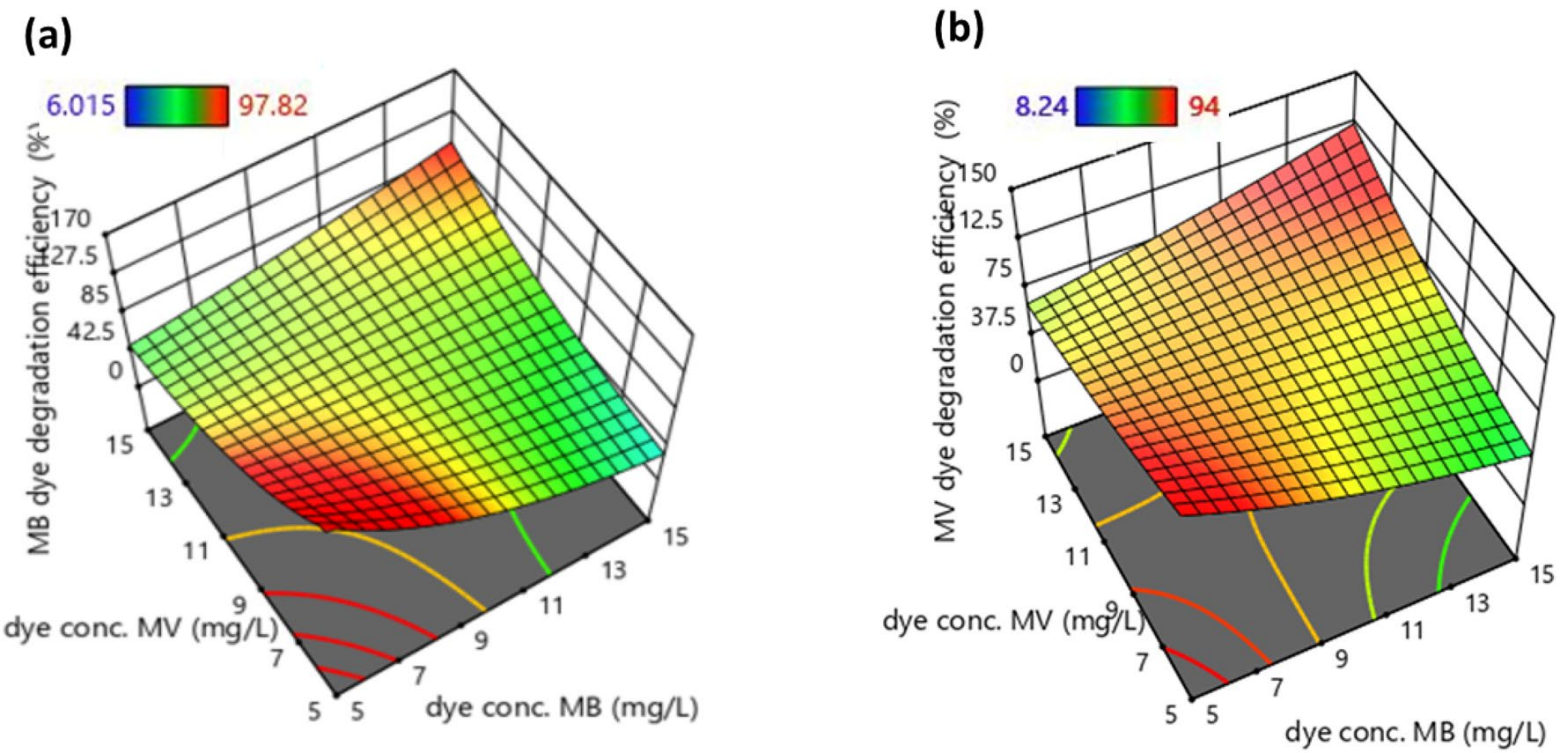
3D plots showing the influence of concentrations of dyes on (**a**) MB dye and (**b**) MV dye degradation efficiencies.

**Figure 8:**
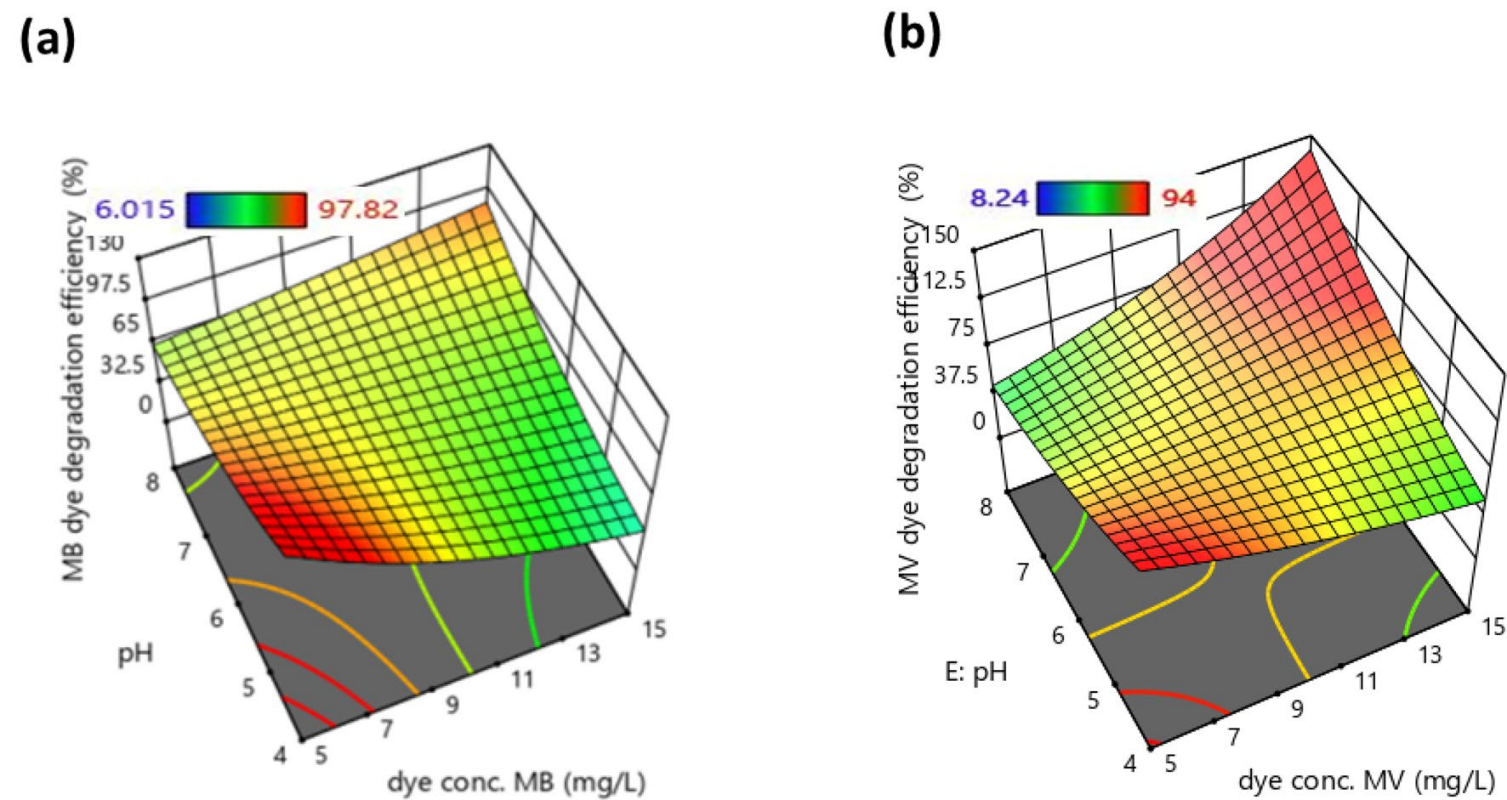
3D plots showing the influence of pH of solution on (**a**) MB dye and (**b**) MV dye degradation efficiencies.

Three‐dimensional response surface plots are used to demonstrate and analyze the combined effects of independent variables. They are also used to identify the major interactions between variables on photocatalytic degradation. The three‐dimensional (3D) surface response was plotted against two factors with other factors being constant (Figs. 6, 7, and 8).

Figure 6 shows the influence of irradiation time and photocatalyst dosage on photocatalytic activity in binary solution. Figure 6a specifically shows the behavior of MB dye degradation efficiency and the influence of these parameters. It is observed that irradiation time has a positive effect on degradation efficiency, which means as much as the irradiation time is high, degradation efficiency will be higher. Furthermore, photocatalyst dosage also has a similar positive effect on photocatalytic activity. Figure 6b shows the influence of photocatalyst dosage and irradiation time on the degradation efficiency of MV dye in binary solution. The influence of the discussed parameters is similar to the case of MB dye degradation efficiency. The positive influence of irrigation time is easily justified because it is similar to photocatalysis reaction time. However, the positive influence of photocatalyst dosage is due to increased surface area promoting the photocatalytic reaction by increasing radical generation^51^. On comparing both plots (Fig. 6 a and b) it is observed that photocatalyst dosage has a higher influence on the degradation activity of MV dye.

Figure 7 shows the influence of individual dye concentrations (MB and MV dye) in binary dye on photocatalytic activity. Figure 7a shows this influence, it can be observed that the MB dye degradation activity in binary solution is high when the individual dyes are very high or very low. Furthermore, when the concentrations of individual dyes are very low, the degradation activity is greater than the activity of a highly concentrated binary solution. Similar types of observations are also seen in the case of individual MV dye degradation efficiency in the binary solution (Fig. 7b). This is because higher concentration leads to more dye molecules. The ratio of reaction sites to dye molecule is low which lowers the degradation efficiency in the predefined time range. Degradation efficiency will be higher if reaction time is high. The ratio of reaction sites and dye molecule is low when the initial concentration is low, which leads to high degradation efficiency^51^ When both are compared it is found that all influences are higher in the case of individual MV dye degradation efficiency in binary solution.

Figure 8, shows the influence of the pH of the dye binary solution. Figure 8a shows that the basicity of the solution supports the degradation activity of individual MB dye in binary solution. Whereas, an acidic solution is good for MV dye degradation activity in binary solution. This is because the compound effect of surface electrical charge characteristics of the catalyst dictates the ionization state of the catalyst surface^52^.

The degradation of dyes was optimized for constraints shown in Table 5. As indicated in the table the degradation activity of both dyes in the binary solution is highly desirable.

**Table 5:** Table of constraints for optimization of photocatalytic degradation of a binary mixture of dye.

Factor	Goal	Lower limit	Upper limit	Lower weight	Upper weight	Importance
X1: Photocatalyst dosage	In range	3	7	1	1	3
X2: Time	In range	75	195	1	1	3
X3: MB dye concentration	In range	5	15	1	1	3
X4: MV dye concentration	In range	5	15	1	1	3
X5: pH	In range	4	8	1	1	3
R1: MB dye degradation efficiency	target = 100	6	100	1	1	5
R2: MV dye degradation efficiency	target = 100	7.8	100	1	1	5

**Figure 9:**
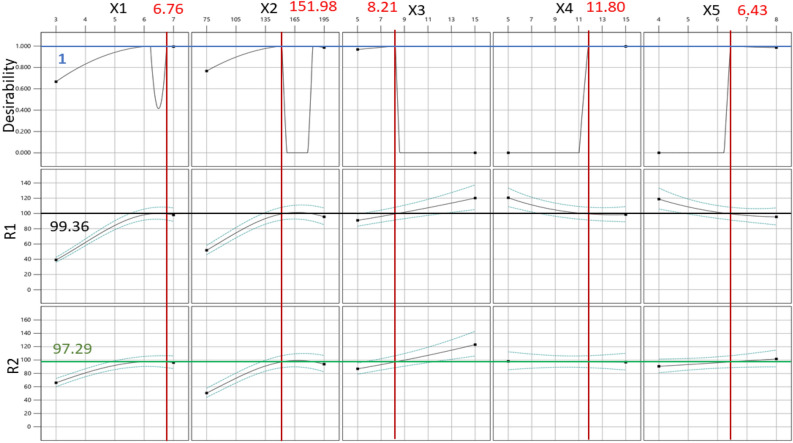
Profiles of desirability and photocatalysis degradation of binary dye mixture.

These constraints and desirable were used to optimize the photocatalytic process through discussed software. This analysis results in Fig. 9. Figure 9 shows profiles of predicated values and desirability functions for degradation of MB and MV dyes in binary solution and indicates the levels of each variable (X1, X2, X3, X4, and X5) in the model and the degradation percentage and desirability function value. The results illustrated in Fig. 8 indicate that the maximum photodegradation of MB and MV using MgO powder as a photocatalyst is 99.36 and 97.29% when 6.76 g/L of catalyst is used for photocatalysis for 152 min while individual concentrates of MB and MV dyes are 8.21 mg/L and 11.8 mg/L respectively moreover the pH of binary solution is 6.43.

**Figure 10:**
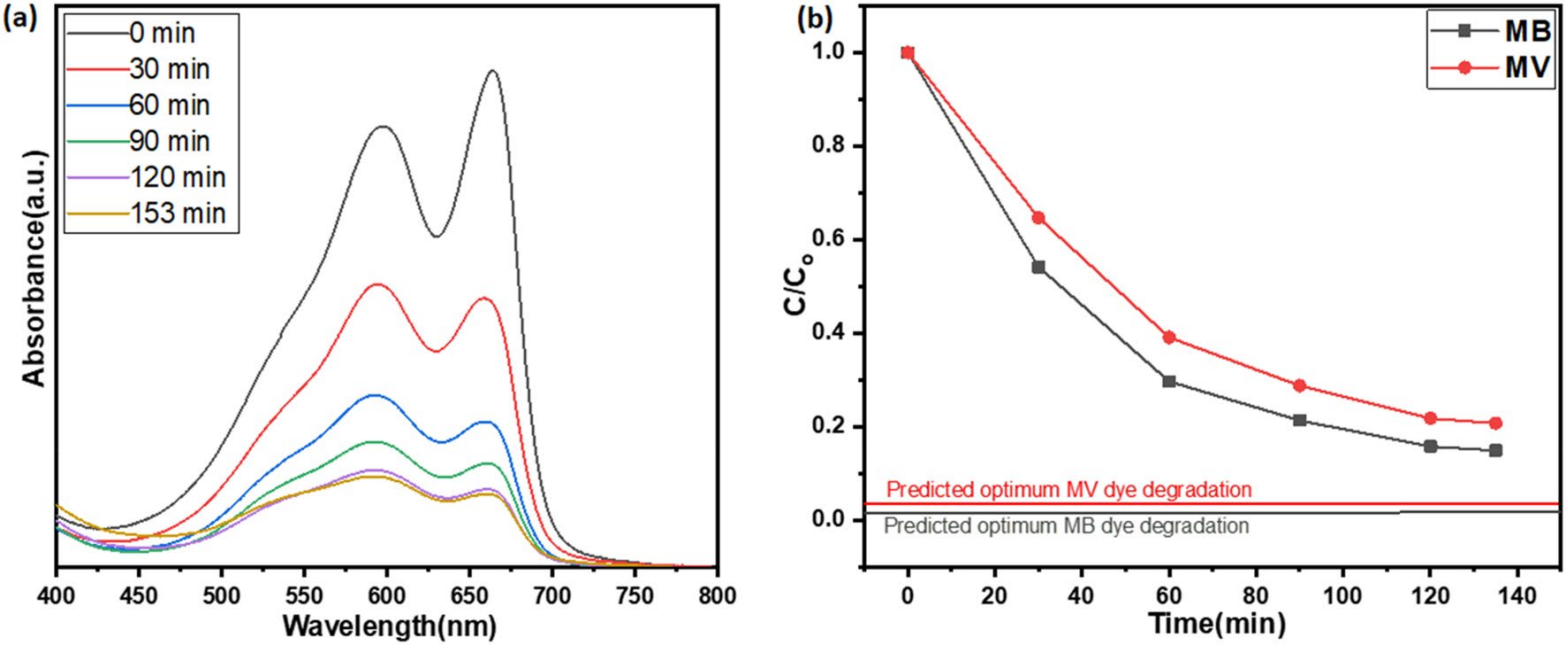
Photodegradation of a mixture of dyes at optimum parameter (**a**) UV- spectra, (**b**) C/C_o_ vs. time.

Optimization study suggested the value of parameters for high degradation efficiency. These values were used for the validation of the optimization study by experiments. The experiments were performed and the results are displayed in Fig. 10. Photocatalytic degradation efficiency on optimum values is 85% and 80% for MB and MV dyes respectively in binary solution. Whereas predicted photocatalytic degradation efficiency is 99.36 and 97.29%. Absorption spectra (Fig. 10a) show that dyes are degradating. Figure 10b shows how MB and MV dyes degrade over time. Maximum photocatalytic degradation efficiency predicated by CCD is marked as a line in Fig. 10b. It can be seen that values are in close agreement with the predicted values. Table 6 shows the comparative study of recently published research articles related to the photocatalysis of textile dye through MgO as a catalyst material. None of the research articles was found related to the binary dye degradation through MgO photocatalyst.

**Table 6:** Comparative study of recently published research articles with present work.

Sr. No	Reference	MgO catalyst modification	Single/binary dye	Time (min)	Degradation activity
1	Naren et al.^53^	Phytochemically enriched MgO nanoparticles	Single dyes: Cationic-congo red dye and Anionic-malachite green dye	120	Congo red dye:95.8% Malachite green dye: 98.5%
2	Kurhade et al.^54^	No modification, MgO nanoparticles, green synthesis, optimization	Single dye: Nigrosine dye	200	96%
3	Kuruthukulangara et al.^55^	No modification, MgO nanoparticles	Single dye: Rhodamine B at pH 12	90	93.5%
4	Shkir et al.^56^	Ag loaded MgO	Single dye: Rhodamine B	120	MgO catalyst: 86%Ag loaded MgO: 91%
5	Barzegar et al.^57^	Phytoextract-mediated synthesis	Single dye: Methylene blue (MB)	120	87%
6	Liu et al.^58^	CuS and MgO loaded with S-doped biochar	Single dye: Rhodamine B	120	95.70%
7	Subalakshmi et al.^59^	Cu_3_(PO_4_)_2_/MgO nanocomposite	Single dye: Amaranth dye	150	98.8%
8	Zhou et al.^60^	Ternary ZnO-Sm_2_O_3_-MgO co-modified biochar	Single dye: Rhodamine B (RhB)	120	99.46%
9	Balakrishnan et al.^61^	MgO nanoparticles	Single dye: Methylene blue (MB)	120	75%
10	Present work	MgO particles	Binary dye: Methylene blue** (**MB) and methylene violet (MV) dye	135	MB dye: 93%MV dye: 88%

## Conclusions

MgO showed high photodegradation efficiency with both MB and MV dyes. The photocatalysis activity of a binary mixture of MB and MV dyes was also examined. MgO shows remarkable photodegradation efficiency of 97% and 88% for MB and MV dye respectively in a binary solution of dyes. The dye mixture degradation reaction follows first-order reaction kinetics according to the L–H model. The influence of process parameters was also evaluated with the help of CCD based on response surface methodology. Five factors (photocatalyst dosage (g/L), time of exposure (min), MB dye initial concentration (mg/L), MV dye initial concentration (mg/L), and pH of the dye solution) were selected for the study. The CCD technique suggested a minimum number of experiments to study the interaction of parameters. The CCD suggested 27 experiments to observe the influence of these parameters on photodegradation. These experiments were performed and the influence was evaluated. Experimental results were fitted to log_10_ transformation of a polynomial to observe the influence. This study suggested that photocatalytic dosage and irradiation time have a positive influence on photocatalytic activity. Whereas the initial concentration of both dyes and pH of binary dye solution has such an influence that the photocatalytic activity is highest at the highest and lowest values. The photocatalytic process was also optimized for maximum photodegradation efficiency by CCD. The CCD suggested optimum values for parameters: photocatalyst dosage of 6.76 g/L, time of exposure of 151 min, MB dye initial concentration of 8.2 mg/L, MV dye initial concentration of 11.80 mg/L, and pH of 6.4. Furthermore, CCD suggested 99.36 and 97.29% of photodegradation efficiencies for MB and MV dyes respectively on optimum parameters. Experiments were also conducted on optimum values suggested by CCD to validate optimization. The validation experiment showed 85 and 80% photodegradation efficiencies for MB and MV dye in binary solution. It can be seen that the values are in close agreement with the predicted values.

## Data Availability

The datasets used and/or analyzed during the current study are available from the corresponding author upon reasonable request.
